# A comparative analysis of genetic variation in rootstocks and scions of old olive trees – a window into the history of olive cultivation practices and past genetic variation

**DOI:** 10.1186/1471-2229-14-146

**Published:** 2014-05-28

**Authors:** Oz Barazani, Erik Westberg, Nir Hanin, Arnon Dag, Zohar Kerem, Yizhar Tugendhaft, Mohammed Hmidat, Thameen Hijawi, Joachim W Kadereit

**Affiliations:** 1 Institute of Plant Sciences, Israel Plant Gene Bank, Agricultural Research Organization, Bet Dagan 50250, Israel; 2 Institut für Spezielle Botanik und Botanischer Garten, Johannes Gutenberg-Universität Mainz, D-55099 Mainz, Germany; 3 Institute of Plant Sciences, Department of Fruit Tree Sciences, Agricultural Research Organization, Gilat Research Center, Gilat, Israel; 4 Institute of Biochemistry, Food Science and Nutrition, Robert H. Smith Faculty of Agriculture, Food and Environment, The Hebrew University of Jerusalem, Rehovot 76100, Israel; 5 Association for Integrated Rural Development (AIRD), Ramallah, Jerusalem Street, Al Nabali Building, P.O.Box 6, Ramallah, The Palestinian Authority

**Keywords:** Domestication, Grafting, Microsatellites, Olive cultivars, Propagation

## Abstract

**Background:**

Past clonal propagation of olive trees is intimately linked to grafting. However, evidence on grafting in ancient trees is scarce, and not much is known about the source of plant material used for rootstocks. Here, the Simple Sequence Repeat (SSR) marker technique was used to study genetic diversity of rootstocks and scions in ancient olive trees from the Levant and its implications for past cultivation of olives. Leaf samples were collected from tree canopies (scions) and shoots growing from the trunk base (suckers). A total of 310 trees were sampled in 32 groves and analyzed with 14 SSR markers.

**Results:**

In 82.7% of the trees in which both scion and suckers could be genotyped, these were genetically different, and thus suckers were interpreted to represent the rootstock of grafted trees. Genetic diversity values were much higher among suckers than among scions, and 194 and 87 multi-locus genotypes (MLGs) were found in the two sample groups, respectively. Only five private alleles were found among scions, but 125 among suckers. A frequency analysis revealed a bimodal distribution of genetic distance among MLGs, indicating the presence of somatic mutations within clones. When assuming that MLGs differing by one mutation are identical, scion and sucker MLGs were grouped in 20 and 147 multi-locus lineages (MLLs). The majority of scions (90.0%) belonged to a single common MLL, whereas 50.5% of the suckers were single-sample MLLs. However, one MLL was specific to suckers and found in 63 (22.6%) of the samples.

**Conclusions:**

Our results provide strong evidence that the majority of olive trees in the study are grafted, that the large majority of scions belong to a single ancient cultivar containing somatic mutations, and that the widespread occurrence of one sucker genotype may imply rootstock selection. For the majority of grafted trees it seems likely that saplings were used as rootstocks; their genetic diversity probably is best explained as the result of a long history of sexual reproduction involving cultivated, feral and wild genotypes.

## Background

Old olive trees in the Levant, estimated to be many hundred years old, are one of the most important component of the rural landscape. Apart from providing edible fruits and valuable storable oil, olive leaves in ancient times were used as fodder for livestock and as a source for paper, stems were used for decoration, different parts of the trees were used in traditional medicine, and fruits and oil were used as offerings in religious ceremonies [1]. Thus, by having high symbolic value and an important place in the ancient literature [1-3], the cultural significance of olive trees is as high as their agricultural and economic value.

Archaeological remains of oil press facilities suggest that cultivation of olive trees in the Levant may have started in the early Bronze Age (ca. 3000 BCE) [4-6]. Genetic analyses suggested that domestication of olive trees may first have started in the Levant [3,7,8] and was later followed by independent domestication across the Mediterranean region [9,10]. Domestication of olives most probably started by selection of wild trees (*Olea europea* subsp. *europea* var. *sylvestris*) with valuable characteristics such as high yield, large fruits, high oil content, etc. that were maintained by vegetative propagation [5]. Following Theophrastos (*De Causis Plantarum* and *Historia Plantarum*, 371–287 BCE), vegetative propagation of olive trees in ancient times included planting of cuttings (leafy stems) and layers (rooting stems that are still attached to the mother tree). Truncheons (hardwood cuttings) were also used, but probably stem knobs (uovuli), which develop at the base of trunks and root easily, were the easiest and most successful propagation technique employed by early growers to propagate desired clones [4].

Although it is unknown when and how grafting was discovered [11], this technique is intimately linked to the history of olive cultivation [4]. It is assumed that grafting of olive trees provided a means to propagate clones which do not root easily, and increased the survival rate of trees, since newly grafted trees required less attention than cuttings [12]. Theophrastos (*HP*, *CP*) provided detailed information on grafting techniques of olive trees in the ancient world, which included crown and bud grafting (summarized by Esler [2] and Foxhall [12]). He also mentioned the use of wild saplings that were dug up and transplanted into groves as rootstocks as a way to generate stronger trees. Recent results support the idea that wild olive trees were used as rootstocks in the Iberian peninsula [13], a technique that has been reported to have been used in the Mediterranean area until recently [12,14], but has increasingly been abandoned with the use of modern techniques for rooting.

Here, we employed the Simple Sequence Repeat (SSR) molecular marker technique to characterize genetic variation in scions and rootstocks of old olive trees from the East Mediterranean. Although detailed information on olive propagation exists from the classical era, molecular evidence for the practice of grafting of olive trees through history is very limited (e.g. [13]). Considering that Israel (IL) and the Palestinian Authority (PA), as one geographical unit, are part of the area in which olive domestication started [3], we assumed that old olive trees in this area may represent an ancient gene pool which can be used for understanding past propagation techniques and the selection of plant material for olive tree cultivation. In particular, we will investigate to what extent the old olive trees studied are the result of grafting, and will look for evidence for the selection of individual genotypes of both scions and rootstocks for olive tree cultivation.

## Results

### Genetic diversity in old olive trees

**Figure 1:**
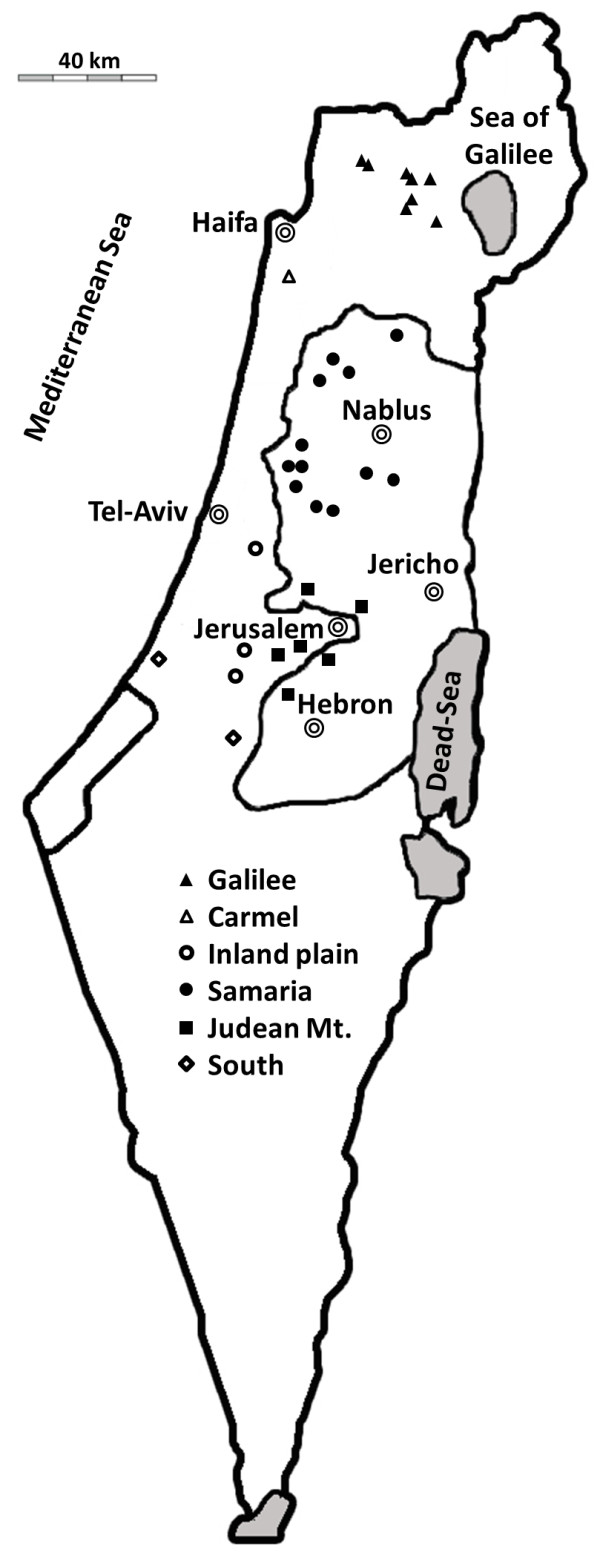
**Location of sampled orchards in Israel and the Palestinian Authority (c.f. Table **1**).**

To investigate the genetic diversity of old olive trees, leaf samples were collected from olive orchards in IL and the PA which we considered to represent ancient groves (Table 1, Figure 1). Fourteen SSR markers were used for genotyping of samples collected from tree canopies and from suckers or shoots from the very base of the trunk. Provided that the trees sampled originated from grafting, these two samples were assumed to represent scions and rootstocks, respectively. Accordingly, in the following we will refer to rootstock and scion as such when the sucker was found to have a genotype different from the canopy of the same tree. A total of 310 trees were sampled for this study. Due to missing data, results are reported for 279 sucker and 280 scion samples. This sample includes data for 249 trees for which both scion and sucker could be sampled. These 249 trees were used for the comparison between scion and sucker genotypes within individual trees.

**Table 1:** **Olive orchards in the different districts of Israel and the Palestinian Authority and number of suckers and scions sampled (c.f. Figure **1**)**

**District**	**# Orchards**		**No. of samples**
		**# Suckers**	**# Scions**
Galilee	8	65	71
Carmel	1	10	8
Inland plain	3	26	21
Samaria	12	110	106
Judean Mt.	6	52	55
South	2	16	19
Total	32	279	280

**Figure 2:**
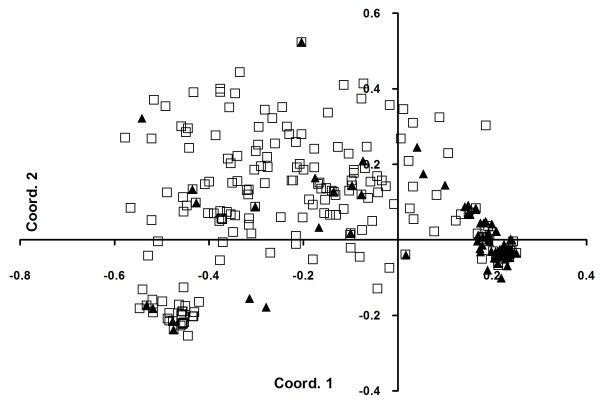
**PCoA analysis of 279 suckers (□) and 280 scions (▲).** The first axis explained 34.46% of the total variation, the second 7.96%.

The number of alleles in the total of 279 sucker and 280 scion samples ranged from five to 28 for the 14 SSR loci (Additional file 1). In general, the average number of alleles was higher in suckers than in scions indicating higher genetic diversity in suckers (Table 2). In addition to this, only five alleles of four different loci were private to scions, whereas 125 alleles of all 14 loci were only found in suckers (Table 2; Additional file 2: Worksheet 2); the majority of private alleles occurred at low frequency. Evidence for higher genetic diversity among suckers than among scions was also obtained by the PCoA analysis in which most scion samples grouped closely together, and where only few samples were more scattered (Figure 2). In contrast, the majority of suckers showed a much more scattered distribution pattern, but some sucker samples grouped with the main cluster of scions (Figure 2).

**Table 2:** Genetic diversity among suckers and scions of old olive trees estimated for the entire sample: Number of different (Na) and effective (Ne) alleles; observed (Ho) and unbiased expected (uHe) heterozygosity; private alleles (Pr. Al.)

	**Na**	**Ne**	**Ho**	**uHe**	**Pr. Al.**
**Suckers**	17.36	4.07	0.78	0.73	125
**Scions**	8.79	2.44	0.80	0.55	5

A total of 258 different multi-locus genotypes (MLGs) were detected among the 559 suckers and scions (Table 3), of which 87 were found in scions and 194 in suckers; of these, 23 MLGs were present in both suckers and scions. Diversity was estimated with one individual per genotype, and most values obtained (Na, Ne, He) were substantially higher in suckers than in scions (Table 2). Observed heterozygosity (Ho) values were high in both suckers and scions, and in addition they were higher than the expected heterozygosity (Table 2). This difference was more pronounced in scions than in suckers (Table 2).

**Table 3:** **Clonal diversity of suckers and scions: Number of multi-locus matched genotypes (MLG), and number of multi-locus lineages (MLL) using a mutational threshold of one; D**_**S **_**represent the corresponding Simpson’s diversity values and R the genotypic richness**

	**Threshold 0**			**Threshold 1**		
	**# MLG**	**D**_**S**_	**R**	**# MLL**	**D**_**S**_	**R**
**Suckers**	194	0.98	0.69	147	0.90	0.53
**Scions**	87	0.86	0.30	20	0.19	0.07

**Figure 3:**
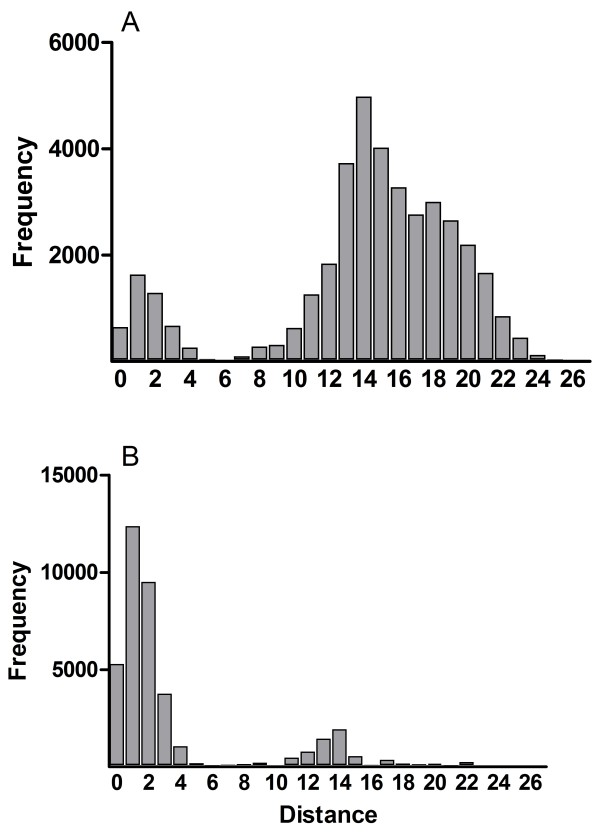
Frequency spectrum of genetic distances among suckers (A) and scions (B).

Because olive trees are propagated vegetatively, we plotted a histogram of pairwise distances to inspect the data for the presence of somatic mutations within clones and to take possible genotyping errors into account. The histogram of pairwise allelic differences among suckers and among scions (Figure 3) showed a bimodal frequency distribution with genotypes differing either by a small or a much larger number of mutational steps. Whereas genotypes differing by a small number of mutational steps are more common among scions, genotypes differing by a larger number of steps are more common among suckers (Figure 3). We assume that genotypes with small differences likely differ due to somatic mutations and possibly genotyping errors, and should be considered part of the same clone or multi-locus lineage. Accordingly, introducing a mutational threshold at which MLGs were grouped together resulted in many MLGs being grouped into multi-locus lineages (MLLs). Raising this threshold from one to five resulted in relatively small differences in the number of different MLLs (Additional file 3) and we henceforth used a threshold of one. This reduced the total number of 258 MLGs to 156 MLLs, of which the total number of MLGs found in scions and suckers was reduced to 20 and 147 MLLs, respectively (Table 3). In accordance with this, clonal diversity estimates were substantially lowered, especially in scions (Table 3).

**Figure 4:**
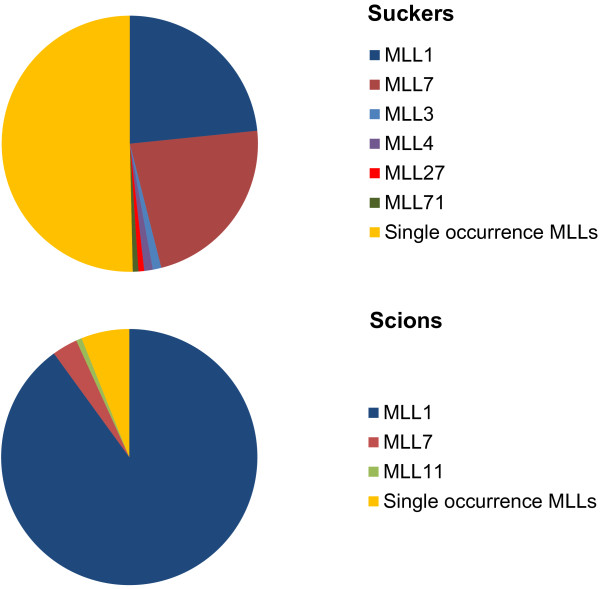
Frequency of multi-locus lineages (MLLs) among 279 suckers and 280 scions.

The frequency of different MLLs among scions and suckers is summarized in Figure 4. In scions, the vast majority of samples (252 of 280) belong to a single MLL (MLL-1). Nine scion samples belonged to MLL-7 which was predominantly found in suckers (Figure 4 and Additional file 2: Worksheet 1). The remaining MLLs among scions were single occurrences (Figure 4), of which nine were also found in the sucker samples of the respective trees. In comparison, 50.5% of the sucker samples (141 of 279) were single sample MLLs (Additional file 2: Worksheet 1). The remaining samples mostly belonged to the common MLL-1 and −7 (65 and 63, respectively). Four sucker MLLs (MLL-3, −4, −26 and −71) were site-specific and were found in two or three suckers from a given grove (Additional file 2: Worksheet 1).

### Genotypic comparison between suckers and scions

In 249 trees both suckers and scions were genotyped (see Additional file 2: Worksheet 3). In 206 of these (82.7%), sucker and scion had different MLGs, while in 43 (17.3%) sucker and scion were identical. In the latter group, 31 trees belonged to the common MLL-1, eight to MLL-7, one was also found in two additional suckers at the same site, and three belonged to single occurrence MLGs (Additional file 2: Worksheet 3). MLL-7 was found only once as a scion grafted on a single occurrence MLL. Accordingly, when sucker and scion had different MLGs they were considered grafted. When identical, they were considered either derived from rooted propagules or self-grafted, which cannot be distinguished from each other.

## Discussion

### Cultivar diversity and cultivation technique

In the 20^th^ Century, an inventory of 27 different olive varieties in former Palestina [15] suggests that in the long history of olive cultivation, cultivars adapted to different regions of the area were selected [16]. Today, local terminology recognizes four cultivars in traditional olive cultivation in the Levant: Souri, Nabali Baladi, Nabali Muhasan and Mallisi. Of these, Souri is the oldest and predominant variety in the region [17]. On the background of these reports of high olive cultivar diversity in the Levant, our results are unexpected. We obtained strong evidence that the overwhelming majority of old olive trees in IL and the PA originate from vegetative propagation of a single ancestral clone. Of the old olive trees analyzed, scions of 252 trees were assigned to MLL-1 (Figure 4; Additional file 2); the bimodal frequency distribution of genetic distances among scions (Figure 3B) suggests that much of the diversity found among scions (Table 2) is due to somatic mutations. This implies that the substantial genetic diversity in the one dominant clone has accumulated during its probably very long existence, as had also been suggested for other ancient olive cultivars [13,18]. Although the diversity found in MLL-1 may be indicative of the antiquity of this ancestral clone, we unfortunately cannot even estimate the age of the trees, as in most cases the old inner parts of the trunks have disintegrated and are not available for radiocarbon dating or dendrochronological analysis. Also, dendrochronology has been shown to be an unreliable method for estimating the age of olive trees [19,20].

The discrepancy between reported cultivar diversity on the one hand [1,15] and absence of proportional variation of microsatellites on the other hand can have two explanations: First, cultivar diversity need not necessarily be reflected in genetic diversity as revealed by the microsatellites used by us. Second, the majority of sampled trees (i.e., those that belonged to MLL-1 in the scions) belong to only one cultivar. Most of the microsatellite markers used by us were also applied in a recent study and successfully differentiated the East Mediterranean Souri from other olive cultivars from around the Mediterranean (e.g., Picual, Koroneiki, Arbequina, Kalamata and others) [21]. Thus, we can discard the first of our two possible explanations and assume that MLL-1 likely represents the most common Souri cultivar [17]; indeed a specimen considered to represent the Souri cultivar was found to belong to MLL-1 (Tugendhaft et al. unpubl. results). However, as our analysis revealed an additional 19 MLLs, besides the one dominant one, most of them as single occurrences (Table 3, Figure 4 and Additional file 2), it is possible that additional cultivars may be hidden among these MLLs. However, considering that a study by Lavee et al. [17] revealed substantial phenotypic and genotypic polymorphism among 14 accessions presumed to belong to the Souri cultivar, it is equally possible that the additional MLLs found by us do not represent different cultivars but rather illustrate that the Souri cultivar is genetically variable and ill-defined.

In the majority of trees (82.7%) in our study, suckers and scions did not share the same MLG, suggesting that these trees were grafted. There are, however, some possible sources of error in the estimation of the frequency of grafted trees: First, it is possible that somatic mutations have occurred within some individuals. This could have resulted in slightly different MLGs in scion and sucker samples of rooted trees, which would have led them to be classified as grafted. Second, considering the high frequency of MLL-1 and MLL-7, there is a high likelihood of sampling grafted trees with an identical MLG in sucker and scion. In consequence, some of our results involving identical and closely related MLGs are likely to be erroneously classified as grafted or non-grafted trees.

**Figure 5:**
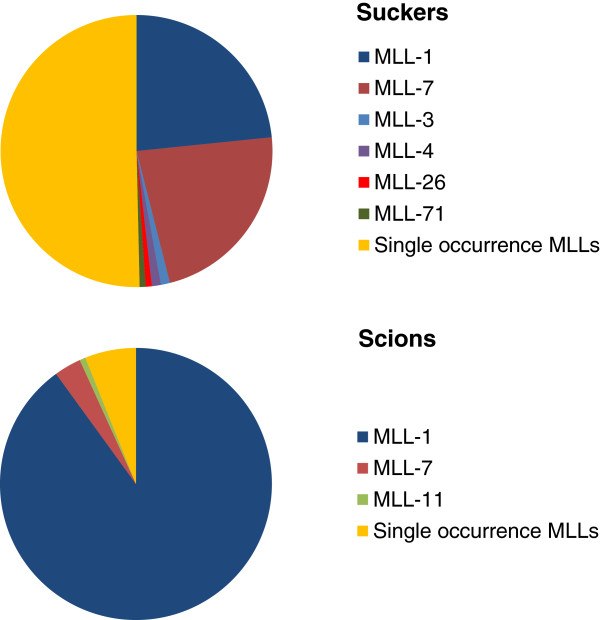
Traditional grafting of olive branches on suckers.

Irrespective of this, our results provide strong evidence that grafting was the most common technique for olive propagation in the Levant at the time when these trees were first grown. To our knowledge, the study by Diez et al. [13] is the only published account of the genetic relationship between rootstocks and scions, but in their study of old olive trees in the Iberian Peninsula only one third of the trees were grafted. Their findings also indicated that grafting was more common in older than in younger trees (estimated by trunk diameter) and in particular cultivars. In our analysis the scions of most grafted trees belonged to MLL-1 (163 trees; Additional file 2: Worksheet 3). It is likely, as argued above, that MLL-1 belongs to the Souri cultivar which does not root easily from leafy cuttings without application of phytohormones. If this is correct, it is plausible to assume that grafting was the easiest way of propagation of the trees sampled in our study. However, in 43 trees, the sucker sample was genetically identical with the scion sample (Additional file 2: Worksheet 3), and 31 of these trees belonged to MLL-1 (Additional file 2: Worksheet 1). It is possible that the genetic identity of sucker and scion in these trees may be the result of sampling mistakes, i.e., samples from suckers of these trees were sampled above the grafting point and thus represent scions rather than rootstocks. However, since we found the rootstock-specific MLL-7 in both scion and sucker of the same individual tree, and vegetative propagation from knobs, cuttings, truncheons and layers was in use in ancient times [4,12], it is also reasonable to conclude that these trees were not grafted. Alternatively, grafting scions on suckers of the same individual may have been used as an easy propagation technique which is still being practiced by some traditional olive growers in the Levant today (Figure 5).

### Genetic diversity in scions and rootstocks

Our findings show that genetic variation among rootstocks is substantially higher than that found among scions (Figure 2; Tables 2 and 3; Additional file 2). The high genetic diversity of rootstocks raises the question about their origin. For this, two possibilities can be considered: First, as postulated for the Iberian Peninsula [13], rootstock variation may represent wild olives, which assumes that wild var. *sylvestris* was common in the Levant [4,5]. Second, the bimodal frequency distribution of genetic distances (Figure 3) indicates that the majority of rootstocks are the result of sexual reproduction. Thus, it is conceivable that scions were grafted on young olive trees which either were germinated and grown for this purpose from seeds of cultivated trees, or which emerged spontaneously as feral trees in the orchards. As in both cases at least one source of rootstocks would have been cultivated olive trees, our data would imply that genetic variation among cultivated trees, as seen in extant rootstocks and not found among extant scions, was much higher in the past. In addition, our private allele analysis revealed the existence of 125 rootstock-specific alleles (Table 2 and Additional file 2). As many of these alleles can also be found in presumably wild populations of the olive tree in our study area (Barazani et al. unpubl. results), it seems most likely to us that the high genetic diversity found among rootstocks resulted from substantial gene flow and recombination that involved wild, feral and cultivated olive trees.

### Rootstock selection

One of the most surprising results of our analysis is the existence of genotype MLL-7 in 22.6% of the rootstock samples. This genotype was found as rootstock in 55 grafted trees and in eight non-grafted trees; in contrast to this, it was found only once as a scion in a grafted tree, illustrating its predominance as a rootstock. The distribution of this clone in all geographical regions except the South district (Additional file 2: Worksheet 1) supports the assumption that this rootstock was consciously selected and distributed. In support of this, Zohary et al. [4] reported that in Turkey suckers or basal knobs of specific wild individuals are collected and grown in olive orchards as a source for rootstocks.

Historical sources describe the use of wild olive trees as rootstock to increase tree vigour [12]. In more modern times it has been suggested that rootstocks have been selected primarily based on the ease of their propagation [22]. However, it has been demonstrated that specific rootstocks can influence tree size and yield [22], improve tolerance to chlorosis caused by Fe deficiency [23], which can be highly significant in the East Mediterranean calcareous soils [24], and improve tolerance to verticillium wilt [22]. Based on these arguments, our results provide first evidence that not only scions, but also rootstocks were selected in historical times. Moreover, since MLL-7 was most often found in combination with MLL-1 (Additional file 2: Worksheet 3) we may hypothesize that rootstock genotype MLL-7 was selected in order to facilitate propagation by grafting. However, more studies are needed to understand the properties of this unique rootstock and its possible additional effects on the scion.

## Conclusions

Considering that most of our knowledge on olive tree propagation is based on old scripts, our results for the first time unambiguously show that grafting on rootstocks was practiced in the past as the main propagation technique in the Levant. In contrast to our expectation of substantial cultivar variation, our results provide strong evidence that the majority of ancient trees originated from a single ancestral clone. High genetic diversity among suckers that were sampled at the base of tree trunks suggests that saplings that originated from sexual reproduction were the major source of rootstocks. However, as 22.6% of rootstocks belonged to a single MLL, our results provide first evidence on selection of rootstocks in past olive tree cultivation. Given the frequency of somatic mutations in the two common scion and rootstock MLLs, these clones are likely to be of very old origin.

## Methods

### Plant material

The occurrence of traditional rain-fed olive groves was mapped in IL and the PA (Figure 1). Irrespective of cultivar identification, 32 groves with old trees with trunk perimeters that ranged between 100 to 1040 cm (mean 280 cm), were selected for sampling. The largest districts of olive cultivation in IL and PA are Galilee, Samaria and Judean Mts. Accordingly, eight, 12 and six groves were sampled in these three regions, respectively (Table 1). One additional grove was sampled in the Carmel, three in the Inland plain and two in the semi-arid South district (a total of 32 groves) (Table 1, Figure 1). Leaf samples from 310 trees were collected from tree canopies and from suckers or shoots from the very base of the trunk.

### Genetic analysis

DNA was extracted using the Invisorb Plant Mini Kit (Invitek) following the manufacturer’s protocol. Previously published SSR markers [18,25-32] were tested for the presence of genetic variation. Of these, 14 resulted in polymorphic and clear and scorable profiles and were used in this study (Additional file 1). PCR conditions for each marker are presented in the Additional file 2. SSR products were separated at the Center of Genomic Technologies (The Hebrew University of Jerusalem) on an ABI automated sequencer (Applied Biosystems) as a multiplex of several loci labeled with three different fluorescent dyes (6-FAM, NED and HEX; Applied Biosystems). Electropherograms were scored manually using Genmarker 1.75 (SoftGenetics, State College, Pennsylvania, USA). After scoring, samples with missing data were excluded from the data set, resulting in a total of 280 scions and 279 suckers. Of these, both scion and sucker could be sampled and genotyped in 249 trees and these were used for the comparison between scion and sucker genotypes within trees. The remaining analyses were performed on the full data set (i.e., 559 samples).

Multi-locus genotypes (MLGs) were identified using GenAlEx v6.3 [33]. Genetic diversity was analyzed as number of different (Na) and effective (Ne) alleles and observed (Ho) and unbiased expected (uHe) heterozygosity using one representative of each MLG. A principal coordinates analysis (PCoA) was used to visualize genetic diversity among samples derived from scions and suckers. The PCoA was performed on the standardized covariance matrix of genetic distances calculated according to Smouse & Peakall [34] using GenAlEx. Comparison between scions and suckers also included identification of private alleles with GenAlEx.

A histogram of pairwise distances to inspect the data for the presence of somatic mutations was performed using the software GenoType v1.2 [35]. Many of the SSR loci used by us do not appear to mutate in accordance with the stepwise mutation model in our material (not shown). For this reason, the infinite allele model was used in which any allelic state can be reached by one mutational step. Using GenoType, we subsequently tried different mutational thresholds at which MLGs were grouped into multi-locus lineages (MLLs) which we assumed to represent clones. The number of clones and Simpson’s diversity based on MLLs were calculated with GenoDive v1.1 [35]. Genotypic richness (R) was estimated as (N-1)/(G-1), in which N is the sample size and G is the number of MLLs. To determine whether trees were grafted, the MLGs of rootstock and scion samples from the same tree were compared.

## Competing interests

The authors declare that they have no competing interests.

## Authors’ contributions

OB, AD, ZK, TH and JWK conceived this study. AD, ZK and YT mapped olive groves and collected the samples in IL; TH and MH mapped olive groves and collected the samples in the PA. NH did the laboratory work; EW and NH analyzed the data. OB, EW and JWK wrote the manuscript. All co-authors approved submission to BMC Plant Biology.

## Supplementary Material

Additional file 1 SSR markers used, their expected size range, repeated motives and number of alleles found. Click here for file

Additional file 2 **Data on multi-locus lineages (Worksheet 1), private alleles (Worksheet 2) and comparison between suckers and scions (Worksheet 3).** Information on PCR reactions and PCR conditions for each locus is given in Worksheets 4 and 5. Click here for file

Additional file 3 Grouping of different multilocus genotypes (MLG) into multilocus lineages (MLL) as a function of the number of mutational steps separating MLGs for suckers (A) and scions (B). Click here for file
