# Supplementary material for: A comparative analysis of genetic variation in rootstocks and scions of old olive trees – a window into the history of olive cultivation practices and past genetic variation
Source: BMC Plant Biol. 2014 May 28;14:146. doi: 10.1186/1471-2229-14-146 (PMC4049413; doi:10.1186/1471-2229-14-146)
Supplement: Additional file 1 — SSR markers used, their expected size range, repeated motives and number of alleles found. [file 1471-2229-14-146-S1.docx]

**Supplementary Table 1.** SSR markers used, their expected size range, repeated motives and number of alleles found

| **SSR Marker** | **Expected range** | **Repeat motif** | **Reference** | **# Alleles** |
| --- | --- | --- | --- | --- |
| IAS-oli26 | 168-210 | (GA)_14_ | Diaz et al. 2006 | 9 |
| UDO99-011 | 115 | (CT)_7_(CA)_10_(CT)_2_(CA)_2_CT(CA)_2_CT(CA)_9_ | Cipriani et al. 2002 | 9 |
| UDO99-024 | 188 | (CA)_11_(TA)_2_(CA)_4_ | Cipriani et al. 2002 | 11 |
| UDO99-025 | 158 | (AC)_16_(AT)_5_ | Cipriani et al. 2002 | 13 |
| UDO99-043 | 174 | (GT)_12_ | Cipriani et al. 2002 | 26 |
| ssrOeUA-DCA1 | 230 (204–230) | (GA)_22_ | Sefc et al. 2000 | 11 |
| ssrOeUA-DCA3 | 250 (228–250) | (GA)_19_ | Sefc et al. 2000 | 28 |
| ssrOeUA-DCA4 | 136 (128–186) | (GA)_16_ | Sefc et al. 2000 | 21 |
| ssrOeUA-DCA9 | 191 (161–205) | (GA)_23_ | Sefc et al. 2000 | 23 |
| ssrOeUA-DCA11 | 179 (125–161) | (GA)_26_(GGGA)_4_ | Sefc et al. 2000 | 16 |
| sssrOeUA-DCA16 | 178 (120–178) | (GT)_13_(GA)_29_ | Sefc et al. 2000 | 27 |
| ssrOeUA-DCA18 | 178 (168–184) | (CA)_4_CT(CA)_3_(GA)_19_ | Sefc et al. 2000 | 16 |
| PA (ATT)2 | 115–136 | (TAA)_6_ | Saumitou-Laprade et al. 2000 | 5 |
| EMO90 | 184 (180–197) | (CA)_10_ | De la Rosa et al. 2002 | 13 |
