# Supplementary figures and images for: A comparative analysis of genetic variation in rootstocks and scions of old olive trees – a window into the history of olive cultivation practices and past genetic variation
Source: BMC Plant Biol. 2014 May 28;14:146. doi: 10.1186/1471-2229-14-146 (PMC4049413; doi:10.1186/1471-2229-14-146)

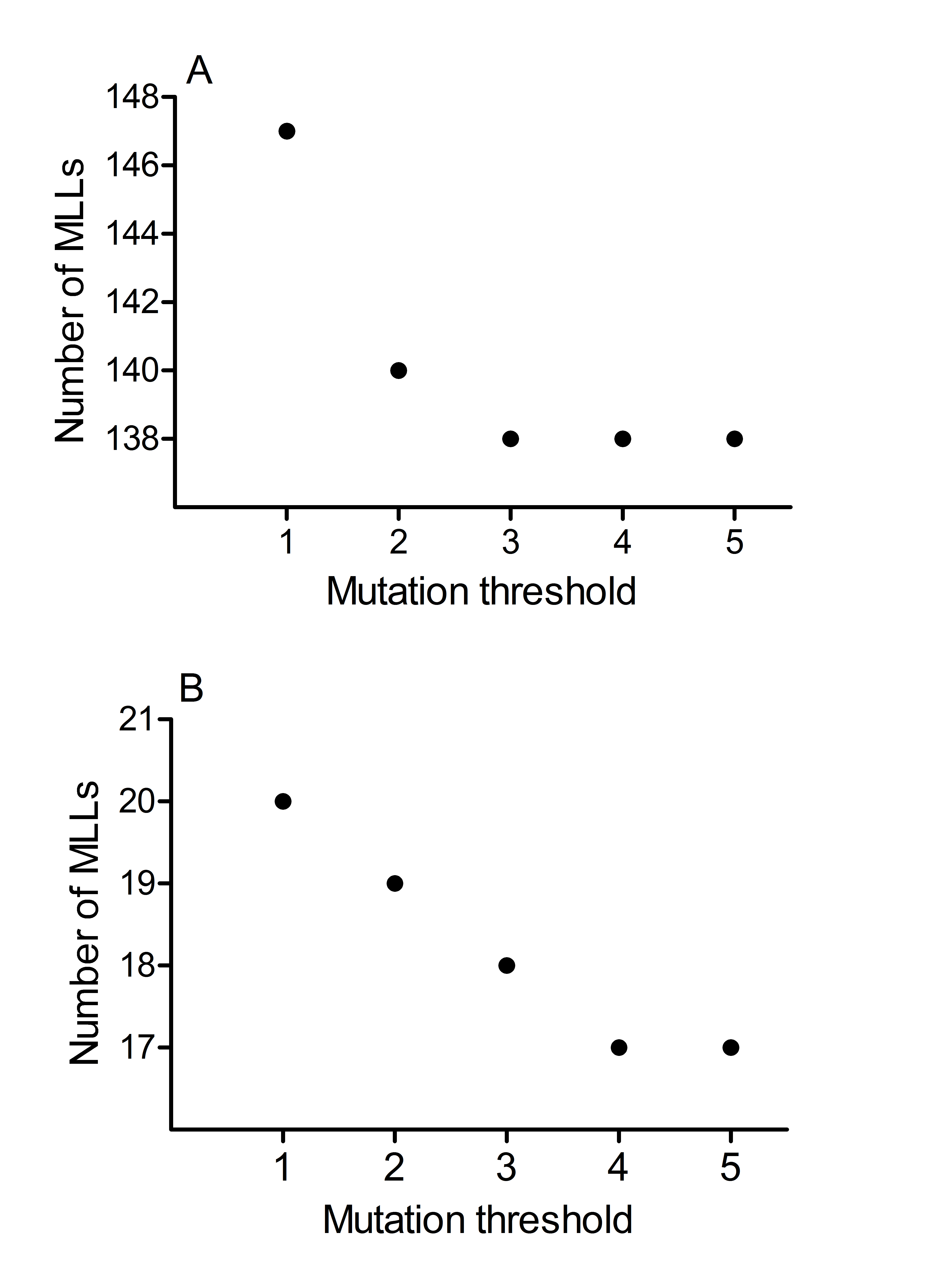

Supplement: Additional file 3 — Grouping of different multilocus genotypes (MLG) into multilocus lineages (MLL) as a function of the number of mutational steps separating MLGs for suckers (A) and scions (B). [file 1471-2229-14-146-S3.jpeg]
